# Which are the clinical characteristics that lead to the decision of primary or secondary usage of TNFi or IL-17i in patients with axial spondyloarthritis?: Real-world evidence from the RABBIT-SpA cohort

**DOI:** 10.1016/j.ero.2025.09.004

**Published:** 2025-10-15

**Authors:** Anne C. Regierer, Anja Weiß, Thomas Marycz, Karin Manger, Martin Bohl-Bühler, Xenofon Baraliakos

**Affiliations:** 1 German Rheumatology Research Center (DRFZ Berlin), Epidemiology and Health Services Research, Berlin, Germany; 2 Praxis für Rheumatologie, Amberg, Germany; 3 Rheumapraxis Manger, Bamberg, Germany; 4 Rheumahaus Potsdam, Potsdam, Germany; 5 Ruhr University Bochum, Rheumazentrum Ruhrgebiet Herne, Germany

## Abstract

**Objectives:**

Assessment of Spondyloarthritis International Society (ASAS)-EULAR treatment recommendations for axial spondyloarthritis (axSpA) suggest advanced therapies after failure of nonsteroidal anti-inflammatory drugs. However, there is insufficient evidence about the best treatment sequence. The primary objective of this study was to analyse the choice of first-/second-line biological disease-modifying antirheumatic drug (bDMARD) treatment as prescribed in routine rheumatology care and to compare patient and disease characteristics at the beginning of first/second bDMARD.

**Methods:**

bDMARD-naïve patients from the prospective German RABBIT-SpA registry initiating their first tumour necrosis factor inhibitor (TNFi) or interleukin-17i (IL-17i) were eligible. Characteristics at beginning of first-/second-line therapy were compared between the 2 groups.

**Results:**

First line: 848 of 1006 patients were treated with TNFi; 158 with IL-17i. Significant differences were found in regard to extramusculoskeletal manifestations (EMMs): psoriasis/uveitis/inflammatory bowel disease (IBD) in 14%/10%/none of IL-17i compared with 7%/14%/5% of TNFi patients. Disease activity, joint count (JC), and patient-reported outcomes were not statistically significant different. Second line: in 289 patients (of those 248 with TNFi, 41 with IL-17i first line), second-line data were available. Of the 248 TNFi first-line patients, 179 (76%) cycled to a second TNFi, whereas 58 (25%) switched to IL-17i. Switchers had significantly higher JC and enthesitis counts. Axial Spondyloarthritis Disease Activity Score did not differ. Significant differences were again found in EMMs: psoriasis/uveitis/IBD in 16%/7%/5% of IL-17i compared with 9%/13%/9% of TNFi patients. Cycler experienced more often secondary failure, switcher more often primary failure in their first-line TNFi.

**Conclusions:**

As recommended in ASAS-EULAR recommendations, EMMs in particular are taken into account in routine care for the treatment decision in first-/second-line bDMARD.

**WHAT IS ALREADY KNOWN ON THIS TOPIC**

• After failure of non-steroidal anti-inflammatory drugs, b/tsDMARDs are indicated in axSpA. However, a personalized approach for the best treatment sequence of first-, second- or further line treatment does not yet exist.

**WHAT THIS STUDY ADDS**

• In our analysis, the main differences in patients treated with first-line or second-line TNFi or IL-17i were found in the presence or absence of extra-musculoskeletal manifestations.

**HOW THIS STUDY MIGHT AFFECT RESEARCH, PRACTICE, OR POLICY**

• To define the best treatment sequence, head-to-head trials and strategic real world studies should stay on the research agenda.

Alt-text: Unlabelled box

## INTRODUCTION

Axial spondyloarthritis (axSpA) is a chronic inflammatory disease of the axial skeleton, leading to severe functional decline, loss of mobility, and reduced quality of life. The management of patients with axSpA relies on nonpharmacological treatments, mainly physiotherapy and regular exercise, and on pharmacological treatments. Then, after failure of nonsteroidal anti-inflammatory drugs (NSAIDs), biologic disease-modifying antirheumatic drugs (bDMARDs) against cytokines such as tumour necrosis factor (TNF) or interleukin-17 (IL-17) and, broader, targeted synthetic (ts) DMARDs with molecules against the Janus Kinase (JAK) pathway are indicated. The recent Assessment of Spondyloarthritis International Society (ASAS)-EULAR treatment recommendations give guidance on the best management based on the available evidence [1]. Although TNFi, IL-17i, and JAKi can be used as first-line b/tsDMARD, it is most common to start with a TNFi or IL-17i because these drugs have the longest evidence on effectiveness and their safety profiles are also described in longer-term follow-ups [2].

With more b/tsDMARDs with different modes of action available, it is necessary to gain evidence on the best treatment sequence. Different aspects need to be taken into account to optimise the sequence in the sense of a personalised approach including patient preferences, comorbidities, and extramusculoskeletal manifestations (EMMs). An evidence-based guideline for an individualised treatment decision about the best first-, second-, or further line treatment does not yet exist. Therefore, physicians in daily practice decide based on each patient’s clinical and safety profile. Also the national health care system might influence this decision. Although in some countries access to recently approved—and thus often very expensive—substances may be restricted due to strict reimbursement rules or delayed market access in the respective country, in Germany market access is often very timely and reimbursement is guaranteed as soon as approval by the national authorities has been granted.

The different b/tsDMARDs have slightly differential effectiveness and safety features especially in regard to EMMs. According to the ASAS-EULAR recommendations, in patients with recurrent acute anterior uveitis or in inflammatory bowel disease (IBD), monoclonal antibodies against TNF are recommended, whereas in patients with severe psoriasis, IL-17i are recommended [1]. IL-17i are contraindicated in the case of active IBD [3]. In the treat to target approach, outcome should be assessed regularly and treatment should be escalated if the target has not been reached.

Based on this knowledge, the aim of this analysis was to describe the choice of first- and second-line bDMARD treatment in patients with axSpA as prescribed in routine rheumatology care in Germany and to compare EMMs, patient and disease characteristics at the beginning of the first and the second bDMARD.

## METHODS

### Data source

The German disease register RABBIT-SpA is a prospective longitudinal observational multicentre cohort study in Germany, which started in 2017. Adult patients diagnosed by the treating rheumatologist with axSpA initiating a new treatment with a bDMARD, tsDMARD, or a conventional treatment (conventional synthetic (cs) DMARDs and/or NSAIDs) can be included into the register upon giving their informed consent. After enrolment, physicians and patients collect data at 3 and 6 months, and then every 6 months. A more detailed description of the study can be found elsewhere [4,5]. RABBIT-SpA received approval by the Ethics Committee of the Charité University Medicine, Berlin (#EA1/246/16). Database closure for this analysis was March 1, 2024.

Data were collected on clinical characteristics, sociodemographics, treatment modalities, and several patient-reported outcomes (PROs). For this analysis, sociodemographic factors included were sex (female/male), age (years), and body mass index (kg/m^2^).

Clinical and disease-specific factors encompassed symptom duration (years), ASAS classification criteria for axSpA fulfilled (yes/no), radiographic status of axSpA (nonradiographic (nr)-/radiographic (r-) axSpA according to modified New York criteria), Spondyloarthritis Research Consortium of Canada (SPARCC) Enthesitis Index (0-16), arthritis (joint count [JC] 0-44), C reactive protein (CRP) (<5 mg/L/≥5 mg/L), HLA-B27 (positive/negative), EMMs history or current including uveitis, psoriasis, and IBD (yes/no). The rheumatic disease comorbidity index, a validated tool to quantify the burden of comorbidities in patients with rheumatic diseases [6,7], was calculated.

Disease activity was measured by the composite Axial Spondyloarthritis Disease Activity Score (ASDAS) calculated with CRP and the patient-reported Bath Ankylosing Spondylitis Disease Activity Index (BASDAI), as well as physician and patient global disease activity on a numerical rating scale (0-10, 10 most severe). Bath Ankylosing Spondylitis Functional Index (BASFI) was assessed to address physical function, ASAS Health Index (ASAS-HI) for global health and functioning, and world health organization-5 well-being index (WHO-5) for depressive symptoms with higher values indicating better mental health status. Patient characteristics and clinical parameters were presented descriptively at beginning of first- and second-line therapy. ASDAS clinically important improvement (ASDAS-CII) was defined as a decrease from start of therapy of ≥1.1 units of the ASDAS until 6 months later.

### Patient selection

In this analysis, bDMARD-naïve axSpA patients who documented their first TNFi or IL-17i since enrolment in RABBIT-SpA were included. Patients treated with tsDMARDs were not included. First-line and second-line therapy episodes with the same active ingredient and with interruption less than 90 days are counted as one episode. The second-line treatment is defined as different substance than the first one or with a time window of more than 90 days between same or different active ingredient. Patients who were not bDMARD naïve, patients with first-line mode of action other than TNFi or IL-17i, and control patients without documented bDMARD were excluded.

### Statistical analysis

Patient characteristics and clinical parameters at beginning of first-line and second-line therapy were presented descriptively separated by treatment line and treatment group. Mean and SD were reported for continuous variables if normally distributed; otherwise, median and IQR were provided. Categorical and binary variables were summarised using frequencies and percentages. Number and percentages of missings are presented in Tables 1 and 2 for every variable. Because of descriptive character of the study, results are presented as observed.

**Table 1:** Demographics and characteristics of study population at initiation of first-line treatment separated by first-line bDMARD

Parameter	Missings n (%)	TNFi n = 848	IL-17i n = 158	Total n = 1006
Age (y), mean (SD)	0	42.5 (13.1)	44.1 (13)	42.8 (13.1)
Females, n (%)	0	371 (44)	64 (41)	435 (43)
Symptom duration (y), median (IQR)	7 (1)	6.5 (11.9)	7.7 (11.1)	6.8 (11.9)
Symptom duration in ≤2 y, n (%)	7 (1)	175 (21)	18 (11)	193 (19)
Diagnostic delay (y), median (IQR)	82 (8)	2.1 (5.6)	2 (6.5)	2 (5.6)
BMI, mean (SD)	26 (3)	26.5 (5.1)	27 (4.8)	26.6 (5.1)
Comorbidities ≥3, n (%)	0	121 (14)	32 (20)	153 (15)
RDCI (0-9), mean (SD)	1 (0)	0.4 (0.8)	0.5 (0.8)	0.4 (0.8)
HLA-B27 positive, n (%)	33 (3)	616 (75)	119 (77)	735 (76)
ASAS classification criteria for axSpA positive, n (%)	0	668 (79)	125 (79)	793 (79)
nr-axSpA, n (%)	577 (57)	104 (29)	31 (41)	135 (31)
CRP ≥ 5 mg/L, n (%)	60 (6)	446 (56)	77 (51)	523 (55)
Enthesitis, n (%)	7 (1)	145 (17)	28 (18)	173 (17)
Enthesitis, number of sites (0-16), mean (SD)	7 (1)	0.4 (1.3)	0.6 (1.5)	0.4 (1.3)
Arthritis, n (%)	5 (0)	248 (29)	42 (27)	290 (29)
Arthritis joint count (0-44), mean (SD)	5 (0)	1.2 (3)	1.2 (3)	1.2 (3)
Physician global disease activity NRS (0-10), mean (SD)	32 (3)	5.6 (1.9)	5.6 (2)	5.6 (1.9)
Patient global disease activity NRS (0-10), mean (SD)	92 (9)	5.8 (2.4)	6 (2.3)	5.8 (2.4)
Patient pain NRS (0-10), mean (SD)	93 (9)	5.5 (2.3)	6 (2.3)	5.6 (2.3)
BASDAI (0-10), mean (SD)	92 (9)	4.6 (1.9)	4.7 (1.9)	4.6 (1.9)
ASDAS, mean (SD)	149 (15)	2.9 (1)	2.9 (1)	2.9 (1)
BASFI (0-10), mean (SD)	92 (9)	3.7 (2.3)	3.8 (2.3)	3.7 (2.3)
ASAS-HI (0-17), mean (SD)	118 (12)	6.7 (3.5)	6.9 (3.5)	6.7 (3.5)
Moderate/severe depressive symptoms, n (%)	28 (3)	212 (28)	41 (29)	253 (28)
Psoriasis, n (%)	23 (2)	61 (7)	22 (14)	83 (8)
Uveitis, n (%)	23 (2)	117 (14)	16 (10)	133 (13)
IBD, n (%)	24 (2)	42 (5)	-	42 (4)
Current NSAIDs, n (%)	5 (0)	601 (71)	113 (72)	714 (71)
Current csDMARDs, n (%)	1 (0)	94 (11)	19 (12)	113 (11)
Current glucocorticoids, n (%)	5 (0)	124 (15)	17 (11)	141 (14)

ASAS-HI, Assessment of Spondyloarthritis International Society Health Index; ASDAS, Axial Spondyloarthritis Disease Activity Score; axSpA, axial spondyloarthritis; BASFI, Bath Ankylosing Spondylitis Functional Index; BMI, body mass index; CRP, c reactive protein; csDMARD, conventional synthetic disease-modifying antirheumatic drug; IBD, inflammatory bowel disease; IL-17i, interleukin-17 inhibitor; NSAID, nonsteroidal anti-inflammatory drug; NRS, numerical rating scale; RDCI: rheumatic disease comorbidity index; TNFi, tumour necrosis factor inhibitor.

**Table 2:** Demographics and characteristics of study population separated by second-line bDMARD

Parameter	Missings n (%)	TNFi→TNFi n = 179	TNFi→IL-17i n = 58	Total n = 237
Age (y), mean (SD)	0 (0)	44.4 (12.5)	49.9 (12.6)	45.8 (12.7)
Females, n (%)	0 (0)	90 (50)	28 (48)	118 (50)
BMI, mean (SD)	29 (12)	26.7 (5)	27.8 (5.3)	27 (5.1)
HLA-B27 positive, n (%)	7 (3)	122 (70)	35 (64)	157 (68)
ASAS classification criteria for axSpA positive, n (%)	0 (0)	143 (80)	38 (66)	181 (76)
nr-axSpA, n (%)	134 (57)	15 (19)	8 (36)	23 (22)
CRP ≥5 mg/L, n (%)	13 (5)	36 (21)	13 (24)	49 (22)
Enthesitis, n (%)	1 (0)	8 (4)	9 (16)	17 (7)
Enthesitis, number of sites (0-16), mean (SD)	1 (0)	0.1 (0.4)	0.5 (1.5)	0.2 (0.8)
Arthritis, n (%)	1 (0)	29 (16)	21 (36)	50 (21)
Arthritis joint count (0-44), mean (SD)	1 (0)	0.6 (1.6)	1.5 (2.9)	0.8 (2.1)
Physician global disease activity NRS (0-10), mean (SD)	10 (4)	3.1 (2.4)	3.4 (2.4)	3.2 (2.4)
Patient global disease activity NRS (0-10), mean (SD)	37 (16)	4.4 (2.8)	5 (2.8)	4.6 (2.8)
Patient pain NRS (0-10), mean (SD)	37 (16)	4.4 (2.7)	5.1 (2.9)	4.6 (2.8)
BASDAI (0-10), mean (SD)	37 (16)	3.7 (2.2)	4.1 (2.1)	3.8 (2.2)
ASDAS, mean (SD)	49 (21)	1.9 (1)	2.2 (1)	2 (1)
BASFI (0-10), mean (SD)	37 (16)	3.2 (2.3)	3.6 (2.7)	3.3 (2.4)
ASAS-HI (0-17), mean (SD)	42 (18)	5.9 (4.1)	6.9 (4.4)	6.2 (4.2)
Moderate/severe depressive symptoms, n (%)	11 (6)	36 (26)	15 (30)	51 (27)
Psoriasis, n (%)	12 (4)	16 (9)	9 (16)	25 (11)
Uveitis, n (%)	8 (3)	22 (13)	4 (7)	26 (11)
IBD, n (%)	11 (5)	15 (9)	3 (5)	18 (8)
Satisfaction with the effectiveness of previous treatment, n (%)	13 (10)	68 (71)	17 (74)	85 (71)
Satisfaction with the tolerability of previous treatment, n (%)	0	75 (79)	19 (86)	94 (80)
Current NSAIDs, n (%)	22 (8)	98 (58)	27 (51)	125 (58)
Current csDMARDs, n (%)	1 (0)	23 (13)	11 (19)	34 (14)
Current glucocorticoids, n (%)	5 (2)	17 (10)	3 (5)	20 (9)

ASDAS, Axial Spondyloarthritis Disease Activity Score; ASAS-HI, Assessment of Spondyloarthritis International Society Health Index; axSpA, axial spondyloarthritis; BASFI, Bath Ankylosing Spondylitis Functional Index; BMI, body mass index; CRP, c reactive protein; csDMARD, conventional synthetic disease-modifying antirheumatic drug; IBD, inflammatory bowel disease; IL-17i, interleukin-17 inhibitor; NSAID, nonsteroidal anti-inflammatory drug; NRS, numerical rating scale; TNFi, tumour necrosis factor inhibitor.

To test the hypothesis, that criteria from the recommendations were used to decide on the mode of action (ie, TNFi vs IL17i) of first- and second-line bDMARD, we compared the following variables: ASDAS-CRP, number of sites with enthesitis, arthritis JC, uveitis, psoriasis, and IBD. For the comparison of number of sites with enthesitis and arthritis JC, 95% CIs for difference means were calculated. For binomial parameters like uveitis, psoriasis, and IBD, we have small numbers of success. Wilson score intervals were calculated to compare the TNFi with IL17i groups [8]. They lead to more robust and accurate estimation for the true proportion than the normal approximation. The treatment retention was shown as survival probability in Kaplan-Meier curves and 95% Hall-Wellner confidence bands for the first-line and second-line therapy after TNFi. Calculations were carried out with SAS version 9.4 (SAS Institute, Cary, USA).

## RESULTS

### Included and excluded patients

Of 1850 patients with axSpA included in RABBIT-SpA in the time from May 2017 up to March 2024, 590 patients who were not bDMARD naïve at enrolment in RABBIT-SpA, 243 patients without any bDMARD treatment documented during follow-up in the register, and 11 patients with a different mode of action than TNFi or IL-17i as first-line therapy were excluded from this analysis, resulting in 1006 patients who were included in this analysis.

### Comparison of baseline characteristics at initiation of first-line treatment

**Figure 1:**
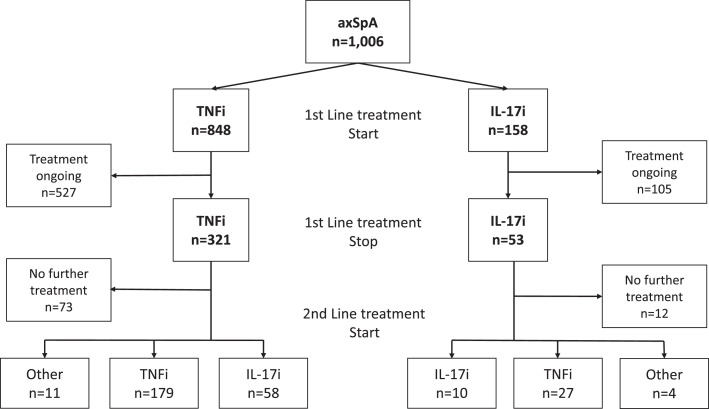
Flow chart showing number of patients with axSpA with their first line and their following second-line treatment. axSpA, axial spondyloarthritis.

Of 1006 included patients, 848 were treated with TNFi and 158 with IL-17i as first-line bDMARD (Fig 1). Adalimumab (55%) was the most used TNFi, followed by golimumab (16%), etanercept (15%), certolizumab (12%), and infliximab (2%). Of the patients with IL-17i as first-line bDMARD, 98% were treated with secukinumab, 2 patients with ixekizumab, and 1 patient with bimekizumab. The patient characteristics at start of first-line therapy are shown in Table 1.

Similarities between patients starting with TNFi and IL-17i were found in the PROs like BASDAI, BASFI, ASAS-HI, pain, and patient global. Furthermore, objective parameters such as the arthritis JC (95% CI [−0.55, 0.48]), sites with enthesitis (95% CI [−0.35, 0.09]) but also the composite score for disease activity ASDAS (95% CI [−0.18, 0.19]) did not show statistically significant differences.

Significant differences between patients starting with TNFi and IL-17i were found in regard to EMMs: psoriasis was present in 14% of IL-17i patients compared with 7% of TNFi patients (95% CI [0.07, 0.11]). In contrast, uveitis was more common in patients initiated with TNFi (14%), as compared with IL-17i (10%) (95% CI [0.12, 0.15]) and IBD was present in 5% of the TNFi-initiated patients but in none of the IL-17i-initiated patients (95% CI [0.03, 0.05]).

### Outcome of first-line treatment at 6-month follow-up and treatment retention

**Figure 2:**
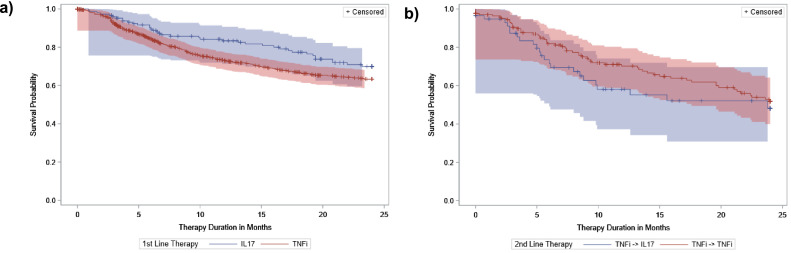
Kaplan-Meier curves of TNFi and IL-17i treatment retention with 95% Hall-Wellner confidence bands of (A) first-line therapy and (B) second-line therapy.

A total of 53% of the TNFi-initiated patients and 42% of the IL-17i-initiated patients experienced ASDAS-CII at 6-month follow-up. Treatment retention for first-line TNFi and IL-17i is shown in Figure 2A. There are no significant differences between the 2 groups. Mean treatment duration of those who stopped first-line treatment was 15.2 months in TNFi and 18.2 months in IL-17i.

We did not find differences in the reasons to discontinue the first-line bDMARD between TNFi and IL-17i-treated patients. Overall, around 50% stopped their first line because of treatment failure, 25% because of side effects, and less than 8% due to remission.

### Comparison of clinical characteristics at initiation of second-line treatment

Of 1006 included patients, a second-line treatment was documented during the follow-up time in 289 patients (Fig 1). Of those, in the first line 248 were treated with TNFi and 41 with IL-17i. Of the 41 patients with first-line IL-17i, in the second line, 10 patients were treated with a second IL-17i and 27 with a TNFi. Because of the small sample size, only the TNFi first-line patients are described further according to their second-line treatment (Table 2). Of the 248 TNFi first-line patients, 179 (76%) continued their treatment with a second TNFi (cycle group), whereas 58 (25%) switched to an IL-17i (switch group).

The patient characteristics at the start of the second-line bDMARD are shown in Table 2. Those who switched to IL-17i showed signs of a higher disease activity than those cycling to TNFi. More patients had peripheral manifestations including arthritis and enthesitis and they had significantly higher arthritis joint (95% CI [-1.52, −0.30]) and enthesitis counts (95% CI [−0.70, −0.22]). The ASDAS did not differ significantly between the 2 groups (95% CI [−0.58, 0.08]). In addition, slightly higher scores in all PROs were found in patients switching to IL-17i as compared with those who stayed on TNFi.

Significant differences between patients cycling to TNFi or switching to IL-17i were found in regard to EMMs: psoriasis was present in 16% of those switching to IL-17i compared with 9% of those cycling to TNFi (95% CI [0.03, 0.08]). Uveitis was more common in those cycling to TNFi (13%), as compared with IL-17i (7%) (95% CI [0.004, 0.03]) and IBD was present in 9% of the TNFi cycler compared with 5% of the switcher to IL17i (95% CI [0.01, 0.08]).

### Second-line treatment groups compared according to outcome characteristics of their first-line treatment and treatment retention

There were differences in the second-line treatment groups in terms of the outcome of their first-line treatment. In the TNFi cycle group, 50% had ASDAS-CII at 6 months after starting their first-line therapy compared with only 22% in those who switched to IL-17i. This means that more patients in the group that cycled to a second TNFi experienced at least temporary treatment benefits during the course of their first TNFi.

Probabilities of drug survival for second-line therapies after a TNFi are shown in Figure 2B. There are no significant differences between TNFi and IL-17i as second-line therapy. Mean treatment duration of first-line TNFi was 15.7 months in the TNFi cycle group compared with 11.2 months in those who switched to IL-17i. In addition, the reasons to discontinue the first-line TNFi were also slightly different: in the cycle group, treatment failure was the reason to discontinue in 51%, and in the switching group it was 72%.

Of those who cycled to a second TNFi, fewer patients experienced (nonserious) adverse events during their first-line TNFi treatment compared with those who switched to IL-17i (53% vs 43% patients, respectively), but more frequently serious adverse events (13% vs 9% patients, respectively).

We did not find differences in the reasons to discontinue the second-line bDMARD between TNFi and IL-17i treated patients. Overall, 53% stopped their second line because of treatment failure, 27% because of side effects, and 3% due to remission.

## DISCUSSION

In axSpA, the optimal treatment sequence, based on objective data for the individualised patient-centred decision, has not yet been defined. Here, we describe the treatment sequence of first- and second-line bDMARDs in a large cohort of patients treated in routine rheumatology care and compare the characteristics at the start of the first-line and the second-line between the main treatment groups. Interestingly, the main differences in patients treated with first-line TNFi or IL-17i were found in the presence or absence of EMMs. At the time point of initiation of the second-line bDMARD, those patients who switched to another mode of action, ie, from TNFi to IL-17i, showed markers of higher disease activity and they had experienced more often a primary failure of their first-line TNFi compared with those cycling to the second TNFi who more often had experienced a temporary benefit and then a secondary failure of their first-line TNFi.

Many patients experience treatment failure of the first-line bDMARD treatment. Therefore, second- and further line treatment options are necessary. An evidence-based guideline for an individualised treatment decision about the best second or further line treatment does not yet exist. However, there is preliminary evidence, that a second-line bDMARD is less effective after TNFi first-line treatment in axSpA [[9], [10], [11]]. This can also be seen in drug retention as a proxy for effectiveness and safety, which is associated with the line of treatment [[12], [13], [14]], which aligns well with our results showing lower retention in the second line, although we did not formally compare first- and second-line treatment. Evidence regarding third line is even less frequent. Effectiveness of second- or third-line TNFi has been demonstrated in a recent analysis of the Euro-SpA research network. More than 70% of the patients were still on their second- or third-line TNFi after 1 year. The rates for inactive disease were 23% in second line and 16% in third line [13].

There is no head-to-head evidence for the best personalised treatment sequence from clinical trial data so far. Currently, there is an ongoing clinical trial with the aim to compare the efficacy of the 2 treatment strategies after a first TNFi treatment failure, either to cycle to a second TNFi or to switch to an IL-17i [15].

In addition, there is only sparse and insufficient evidence from observational data on this research question. An analysis of the Swiss Clinical Quality Management registry compared effectiveness of IL-17i with TNFi in patients with axSpA with prior exposure to TNFi. Drug retention and BASDAI 50 response was very similar between the 2 treatment groups [16]. In an analysis from real-world data from Germany, drug retention was higher in TNFi compared with IL-17i and JAKi, with more first-line treatments in the TNFi group [17]. In an analysis of more than 10,000 treatment episodes from 5 Scandinavian disease registries, the treatment effectiveness of IL-17i was compared with TNFi. Drug retention was similar, but IL-17i was only rarely used as first line in that study [14.

An unicentric analysis of 335 axSpA patients from the Netherlands compared the second-line strategy after TNFi first-line treatment [18]. The treatment strategy was either to cycle from first-line TNFi to a second-line TNFi or to switch from TNFi to an IL-17i. This was dependent on the time point, as these strategies were based on local treatment protocols that were changed in December 2019. The authors described a significantly higher drug retention rate for the cycle strategy (hence the earlier time period from the years 2012-2019) compared with the switch strategy (from 2019-2023). However, from our point of view, it is very likely that the treatment time point has a strong impact on the drug retention rate. That means, it is very probable that the drug retention was longer in their analysis in the first time period because at that time there were fewer options to change the treatment to.

Although there is no clear evidence on the best treatment sequence, there are some aspects that should be considered for the personalised choice of bDMARD [1,2,19]. The ASAS-EULAR recommendations state that the patients‘ preference, EMMs, comorbidities, and safety/intolerance of certain drugs should be taken into account for treatment escalation from NSAIDs to a b/tsDMARD [1]. Interestingly, this pattern can be found in our data in regard to the clinical characteristics at the time point of start of the first-line treatment. Although patients with uveitis and IBD were more often treated with TNFi, patients with psoriasis were more often treated with IL-17i.

The clinical characteristics in our cohort at the time point of start of the second-line treatment shows some interesting details as well. The patients switching to IL-17i showed a more severe disease activity with higher enthesitis and arthritis counts than those cycling to a second TNFi. There were also differences in the course of their first-line TNFi treatment. The patients in the TNFi cycle group had a better outcome of their first-line treatment with a higher rate of ASDAS-CII and a longer treatment duration. This means that patients who experienced secondary loss of efficacy with their first TNFi were more likely to receive a second TNFi as the second bDMARD, whereas patients with primary loss of efficacy were more likely to switch to a different mode of action (ie, from TNFi to an IL17i). Therefore, the results of our analysis reflect the personalised approach by the ASAS-EULAR recommendations to take into account the previous experience of the bDMARD first-line treatment.

Our analysis has limitations. In the second-line treatment groups, the numbers of included patients are low; hence, we were not able to look into the sequence with first-line IL-17i and then the switch to TNFi as second line. Because of the observational design of our analysis, there might be several potential confounders that can affect the treatment choice and also the outcome of the respective treatment.

## CONCLUSION

As recommended in the ASAS-EULAR treatment recommendations, the EMMs in particular are taken into account in routine care for the treatment decision in first- and second-line bDMARD. At the time point of initiation of the second-line bDMARD, those patients who switched to another mode of action, ie, from TNFi to IL-17i, showed markers of higher disease activity and they had experienced more often a primary failure of their first-line TNFi compared with those cycling to the second TNFi who more often had experienced a temporary benefit and then a secondary failure of their first-line TNFi.

## Editor disclosure

The peer review process did not involve Editorial Board Member Xenofon Baraliakos, and the editorial decision making was led by the editors not involved in the creation of this manuscript.

## CRediT authorship contribution statement

**Anne C. Regierer:** Writing – review & editing, Writing – original draft, Visualization, Methodology, Funding acquisition, Conceptualization. **Anja Weiß:** Writing – review & editing, Writing – original draft, Visualization, Software, Methodology, Formal analysis, Data curation. **Thomas Marycz:** Writing – review & editing, Resources. **Karin Manger:** Writing – review & editing, Resources. **Martin Bohl-Bühler:** Writing – review & editing, Resources. **Xenofon Baraliakos:** Writing – review & editing, Writing – original draft, Supervision, Resources, Conceptualization.

## Competing interests

All authors declare no conflicts of interest.
